# Evaluating the effect of gamma rays on *Zamiifolia (Zamioculcas zamiifolia*) plant in vitro and genetic diversity of the resulting genotypes using the ISSR marker

**DOI:** 10.1038/s41598-023-35618-2

**Published:** 2023-05-23

**Authors:** Ebrahim Beyramizadeh, Ali Arminian, Arash Fazeli

**Affiliations:** 1 Ornamental Plants Research Center (OPRC), Agricultural Research, Education and Extension Organization (AREEO), Horticultural Sciences Research Institute (HSRI), Mahallat, Iran; 2 Former Ph.D. student, Agronomy and Plant Breeding Department, Agricultural Faculty, Ilam University, Ilam, Iran; 3 Agronomy and Plant Breeding Department, Agricultural Faculty, Ilam University, Ilam, Iran

**Keywords:** Plant biotechnology, Plant breeding, Plant evolution, Plant genetics, Plant breeding

## Abstract

*Zamiifolia* (*Zamioculcas sp.*) is a perennial plant in the *Araceae* family and one of the new apartment plants in the world. In this study, in order to increase the effectiveness of the breeding program, tissue culture technique and explants of leaf parts were used. The results indicated that 2,4-D (1 mg/l) and BA (2 mg/l) hormones affected positively and significantly callus formation and simultaneous application of NAA and BA (both in 0.5 mg/l) caused the best results regarding seedling production and number, leaves, complete tubers, and root in tissue culture of *Zaamifolia*. In the study, three cultivars of *Zamiifolia* (green, black and Dutch) and 12 genotypes resulted from callus formation stage, irradiated with different gamma rays (0 to 175 Gy, with LD_50_ as 68 Gy) were selected and the presence of genetic diversity was investigated using 22 ISSR primers. Applying ISSR marker showed that the highest amount of PIC values related to the F19(0.47) and F20(0.38) primers, which persuasively isolated the studied genotypes. Moreover, the highest efficiency was detected for AK66 marker based on the MI parameter. The PCA and clustering categorization via UPGMA methodology based on molecular information and Dice index, differentiated the genotypes into 6 groups. Genotypes 1(callus), 2(100 Gy) and 3(cultivar from Holland) created separated groups. The 4th group included 6(callus), 8(0 Gy), 9(75 Gy), 11(90 Gy), 12(100 Gy) and 13(120 Gy) genotypes appearing as the largest group. The 5th group included 7(160 Gy), 10(80 Gy), 14(140 Gy) and 15(Zanziber gem black) genotypes. The last group included 4(mather plant) and 5(callus) genotypes. In this context, genotypes 1, 5, and 6 had probably somaclonal variation. Moreover, genotypes that received doses of 100 and 120 Gy, had a medium diversity. There is a high possibility of introducing a cultivar with a low dose and high genetic diversity in the whole group. Genotype 7 in this classification, received the highest dose of 160 Gy. In this population, the Dutch variety, was used as a new variety. As a result, the ISSR marker could correctly group the genotypes. This is an interesting finding, and it could be hypothesized that the ISSR marker could correctly differentiate *Zaamifolia* genotypes and probably other ornamental plants under the effect of gamma rays mutagenesis in order to achieve novel variants.

## Introduction

Zamiifolia (*Zamioculcas Zamiifolia*) commonly named as: aroid palm, aroid plant, emerald palm, eternity plant, Zanzibar gem, Zuzu plant, ZZ plant is one of the important houseplants in the *Araceae* family with relatively narrow glossy green or black leaves. This plant has thick horizontal rhizomes which can grow in low light conditions and is highly tolerant to drought stress, diseases, and pests^1^. Nowadays, plant tissue culture techniques have significantly increased the efficiency of breeding methods, including breeding using mutagenesis. The use of mutation induction methods, e.g. by means of gamma rays, in ornamental plants is promising^2^. Some of the main traits in annual ornamental plants obtained by mutation induction are flower color, flower increase, flower shape, leaf shape, number of flower petals, leaf size, plant size, plant smallness, large flowers, form plant, growth habit, growth rate, number of branches and flower longevity^3^. At the species and genus level, *Zamiifolia* does not have a natural variety of flowers and lacks cross breeding and hybridization. Breeding by mutation, therefore, is a logical solution to create *Zamiifolia* with desired genes in its genome.

Heping and Peng^4^ evaluated micropropagation of *Zamiifolia* plant from the original leaf sample in the culture medium of Murashige and Skoog^5^ (MS) containing 20 mg/l benzyl adenine (BA) and 0.02 mg/l Alpha-naphthalene acetic acid (NAA) for stem formation. The rooting of stems was done in MS medium containing 0.5 mg/l litrandol-3-butyric acid (IBA). Vanzie-Canton and Leonhardt^6^ examined the *Zamiifolia* plant tissue culture using sepal and leaflet tissue. A 1/2 MS medium with 100 mg/l myoinositol, 0.2 mg/l benzyl adenine BA, 4 mg/l 2,4-dichlorophenoxyacetic acid (2,4-D), 20 g/l sucrose and 3 g/l agars were used for callus induction. After 4.5 weeks, the cultures were transferred to the stem formation medium, including 1.2 MS medium with 100 mg/l myoinositol, 1 mg/l BA, 40 g/l sucrose, and 3 g/l agars. The mutation rate increases by temperature, long-term storage of seeds, tissue culture conditions, irradiation, and chemical mutagens. Irradiation and chemical mutagens are the most effective methods^7^. Gamma-ray has a wavelength shorter than X-rays, but its energy and penetration power in the tissues is high. The sources of this irradiation are used for irradiating seeds in the form of gamma cells and for irradiating plants in the conditions of small chambers, greenhouses or fields^8^. Most of the desired traits in ornamental plants are visible, and the breeding basis of mutagenesis is based on selecting the desired line using traits. Morphological traits cannot accurately identify cultivars due to the influence of environmental factors. Molecular markers are not affected by environmental conditions or plant developmental stages^9,10^. These markers can be applied to identify ornamental plants, genetic diversity and genome mapping. Simple sequence repeat (ISSR) primers are semi-random markers among the most widely used molecular markers for preparing a genetic map due to their high reproducibility, simplicity, and low cost in studying genetic diversity. In this way, it is not necessary to have information about the genomic sequence to design this marker^11^, and they are mainly known as dominant markers^12^ in which and working with ISSR is easy and fast and they show high level of polymorphisms^13^. Since the ISSR-PCR technique does not require basic knowledge about the genome sequence, it could create many polymorphic and multi-locus patterns. Therefore, the aim of this research is to investigate the creation of genetic diversity by means of 22 ISSR primers in three varieties of *Zamiifolia* (green, black and Dutch*)* and 12 genotypes obtained from the stage of callus formation and irradiation with different doses of ray gamma.

## Results

### Callus induction test

The results of the analysis of variance showed that different concentrations of 2,4-D and BA hormones significantly affected the percentage and volume of callus formation (*P* ≤ 0.05). The results of mean comparison of 2,4-D and BA on percentage and volume callus showed the simultaneous use of 1 mg/l per liter of 2,4-D and 2 mg/l of BA culminated in the best results (Table 1).

**Table 1:** Comparison of means effect of different concentrations of 2,4-D and BA on percentage and volume of *Zamiifolia* callus.

Traits		2,4-D (0 mg/l)	2,4-D (1 mg/l)	2,4-D (2 mg/l)	2,4-D (4 mg/l)
Percentage of callus	BA (0 mg/l)	2.6 ef	5.94 b	4.45 cd	2.29 f.
	BA (1 mg/l)	4.9 bc	0.5 i	3.73 def	5.94 b
	BA (2 mg/l)	4.9 bc	7.76 a	1 j	3.38 cde
Volume of callus	BA (0 mg/l)	1.25 c	1.22 c	1.22 c	1.54 b
	BA (1 mg/l)	1.22 c	0.5 d	1.22 c	1.87 a
	BA (2 mg/l)	1.22 c	1.87 a	0.5 d	1.22 c

Treatments that have at least one letter are not significantly different (*P* > 0.05) according to Duncan's test at 5% probability level.

### Regeneration test

The analysis of variance indicated that different concentrations of NAA and BA significantly affected the percentage of seedling production and the number of leaves, tubers and root. In this research, the simultaneous use of 0.5 mg per liter of NAA and 0.5 mg per liter of BA caused the highest percentage of seedling production and number, leaves, complete tubers, and root (Table 2).

**Table 2:** Comparison of different average concentrations of NAA and BA in *Zamiifolia* regeneration.

Traits		Seedling production percentage	number of leaves	Number of tuber	Number of root
NAA	0	4.32 b	1.36 b	1.25 b	1.25 b
	0.5	6.28 a	1.74 a	1.52 a	1.58 a
BA	0	3.40 b	1.13 b	1.22 b	1.26 b
	0.5	6.37 a	2.63 a	1.63 a	1.59 a
	1	6.10 a	1.88 b	1.31 b	1.39 b

Treatments that have at least one letter are not significantly different (*P* > 0.05) according to Duncan's test at 5% probability level.

### Determination of the optimal intensity of gamma rays

**Figure 1:**
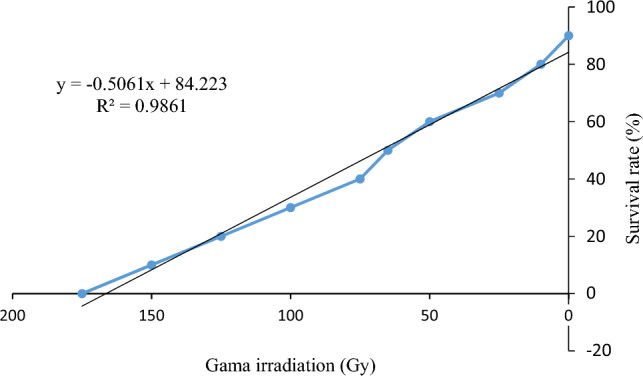
Fitted coefficient of determination for the survival rate of *Zamiifolia* callus.

The irradiation results of *Zamiifolia* callus explants revealed that the survival percentage of the cultivars decreased with the increase of irradiation dose (actually, the use of different doses of mutagen significantly affected the survival percentage and regeneration of *Zamiifolia*. In addition, LD_50_ was calculated using linear regression and the dose was determined as 68 Gy, Fig. 1).

### Molecular markers test results

Absorbance at 230, 260, and 280 nm was measured, and A_260/280_ and A_260/230_ ratios were assessed to determine DNA purity. This ratio should be about 1.8–2 for A_260/280_ and higher than 1.5 for A_260/230_ in a pure DNA sample. The electrophoresis of the DNA extraction product also demonstrated the proper quality and quantity of the extraction. Banding patterns of ISSR markers were scored as one (the presence of a band) and zero (the absence of a band) for each primer and imported into Excel software (Microsoft Excel 2016). The matrix of data obtained from markers was analyzed using GenALEx software adopting dominant markers. In this study, 112 out of 236 amplified bands (47.5%) revealed polymorphism. The amplified bands ranged from 7 to 15 which belonged to CM2 and SD7 primers, respectively. The amount of polymorphism created among the primers was calculated from 25 to 81.81%, where L19 primer showed the least, and AK66 primer showed the most polymorphism, respectively (Table 3). The highest amount of polymorphic information content (PIC) parameter related to the F19 (0.47) and F20 (0.38) primers, which had the most power in isolating the studied genotypes. The lowest amount of PIC was also observed in the AK66 primer (Table 3).

**Table 3:** Genetic diversity parameters related to 22 ISSR primers, including the total amplified band, polymorphic band number, polymorphic percentage, polymorphic information content (PIC), number of different alleles, effective number of alleles, expected heterozygosity, and Shannon information index of *Zamiifolia* genotypes.

	Primer	Total no. of bands	No. of polymorphism bands	Polymorphism percent (%)	Polymorphic information content (PIC)	Na: no. of different alleles	Ne: no. of effective allele	He: expected heterozygosity	uHe: unbiased expected heterozygosity	I: Shannon's information index
1	A86	10	5	50	0.22	1.50	1.25	0.16	0.17	0.25
2	AG5	11	6	54.54	0.16	1.45	1.25	0.16	0.16	0.24
3	AHS	10	5	50	0.14	1.50	1.30	0.17	0.18	0.25
4	AK66	11	9	81.81	0.06	1.18	1.10	0.06	0.06	0.08
5	AN1	12	6	50	0.14	1.50	1.43	0.23	0.24	0.33
6	AVC1	14	4	28.57	0.16	1.71	1.50	0.27	0.28	0.40
7	BCV3	11	5	45.45	0.26	1.55	1.42	0.23	0.24	0.34
8	C07	14	5	35.71	0.22	1.64	1.33	0.20	0.20	0.30
9	C16	9	4	44.44	0.23	1.56	1.45	0.25	0.25	0.35
10	CM2	7	3	42.85	0.27	1.57	1.29	0.19	0.20	0.30
11	F19	9	4	44.44	0.47	1.56	1.50	0.26	0.28	0.37
12	F20	12	5	41.66	0.38	1.58	1.42	0.24	0.25	0.35
13	GN21	10	6	60	0.15	1.40	1.36	0.19	0.20	0.27
14	K1B	10	5	50	0.31	1.50	1.37	0.20	0.21	0.30
15	L19	8	2	25	0.27	1.63	1.33	0.21	0.22	0.33
16	LC26	9	7	77.77	0.1	1.22	1.20	0.10	0.11	0.15
17	OP1	9	4	44.44	0.21	1.56	1.39	0.22	0.22	0.32
18	PD12	9	3	33.33	0.23	1.67	1.50	0.29	0.30	0.41
19	SD7	15	5	33.33	0.23	1.60	1.48	0.26	0.27	0.38
20	SST	13	8	61.53	0.11	1.38	1.34	0.18	0.19	0.25
21	SWE	10	7	70	0.29	1.30	1.18	0.11	0.12	0.17
22	WB7	13	4	30.76	0.24	1.69	1.50	0.28	0.29	0.40
	Total	236	112							
	Average	10.72	5.09	47.98	0.22					

### Cluster analysis

**Figure 2:**
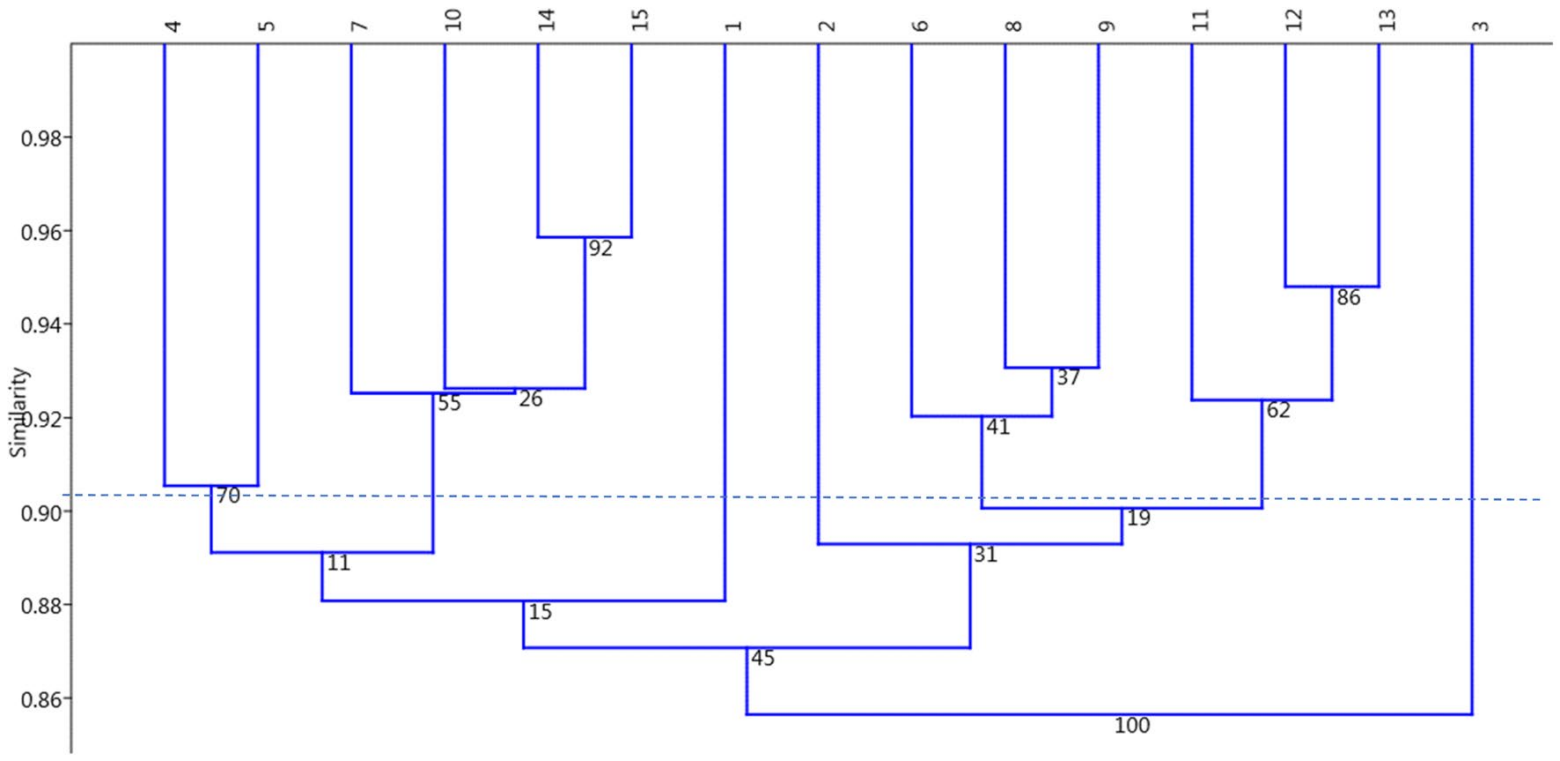
Cluster categorization of 15 *Zamiifolia* genotypes based on ISSR marker band patterns using Dice similarity coefficient and UPGMA algorithm with 1000 bootstraps. The numbers from 1 to 15 on the horizontal axis represent the genotypes codes.

The cluster result of 15 studied genotypes based on ISSR marker band patterns using the Dice similarity index^14^  and UPGMA algorithm through SAHN algorithm, as defined by Sneath and Sokal^15^ represented in Fig. 2. According to the dendrogram, it could be seen that the genotypes were classified into 6 groups: Genotypes 1(callus), 2(100 Gy) and 3(cultivar from Holand) created separated groups. The 4th group included 6(callus), 8(0 Gy), 9(75 Gy), 11(90 Gy), 12(100 Gy) and 13(120 Gy) genotypes as the largest group. The 5th group included 7(160 Gy), 10(80 Gy), 14(140 Gy) and 15(Zanziber gem black) genotypes. The last group included 4(mather plant) and 5(callus) genotypes. In this context, genotypes 1, 5, and 6 had probably somaclonal variation. Moreover, genotypes that received doses of 100 and 120 Gy, had a medium diversity. There is a high possibility of introducing a cultivar with a low dose and high genetic diversity in the whole group. Genotype 7 in this cluster, received the highest dose of 160 Gy.

### Principal component analysis (PCA)

**Figure 3:**
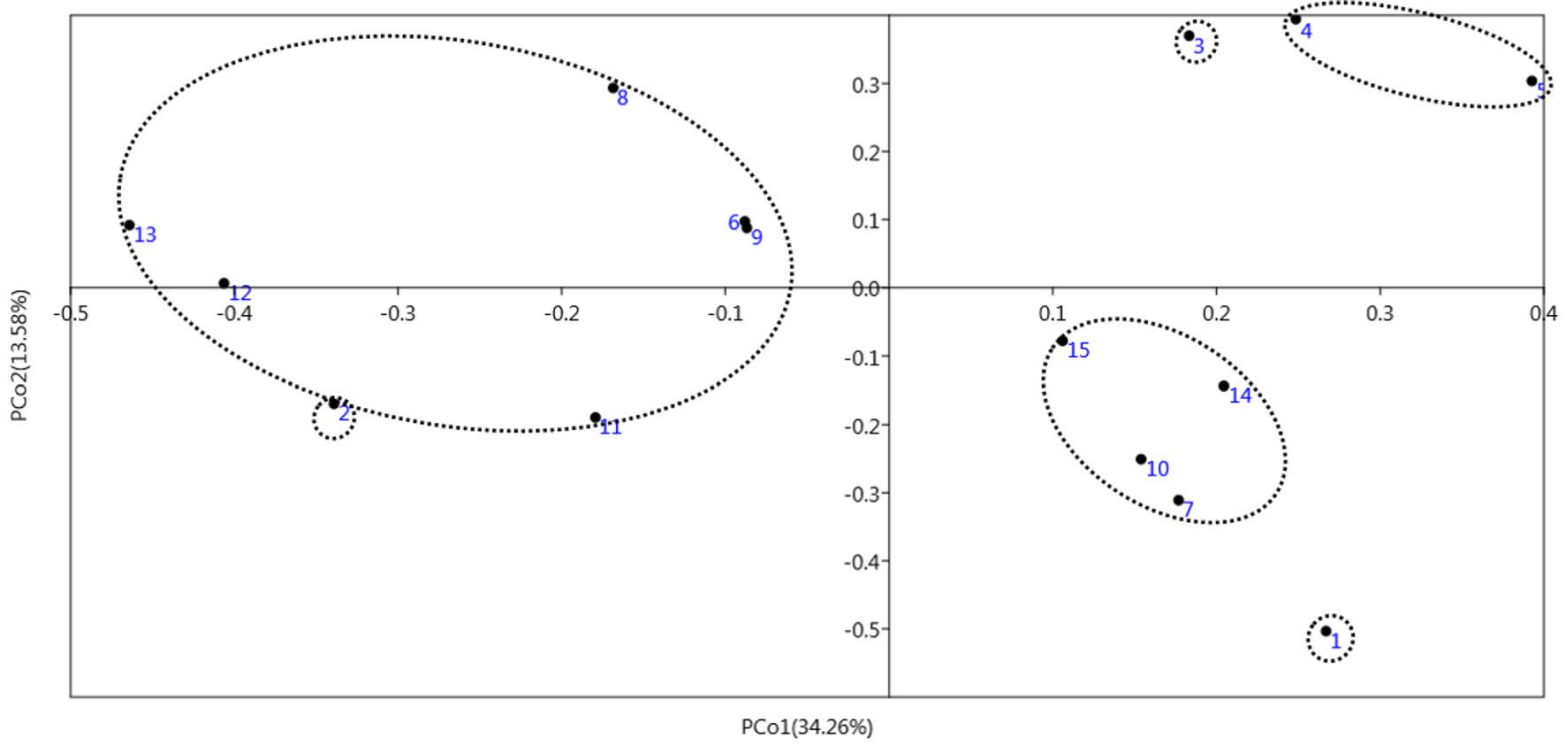
Graphic representation of the classification of *Zamiifolia* genotypes in the principal coordinate analysis.

The principal component analysis (PCA) is generally used to reduce the volume of data to preserve the most effective components of the data set and to better interpret the results in molecular data analysis. In PCA analysis, the first two components related to the ISSR indicator explained 47.83% of the total variation (Table 4). The first five components accounted for a high percentage (77.29%) of the changes at the molecular (Fig. 3). From a genetic point of view, these results indicate a favorable sampling of the entire genome by the markers.

**Table 4:** Principal coordinate analysis of ISSR markers.

Principal coordinate	Eigen value	Variance explained (%)	Cumulative variance explained (%)
PCo1	0.1144	34.256	34.256
PCo2	0.0453	13.577	47.833
PCo3	0.0416	12.465	60.298
PCo4	0.0304	9.095	69.393
PCo5	0.0264	7.900	77.293

## Discussion

The results of the callus induction test revealed that the modified MS basic medium with a concentration of 2 mg/l BA and 1 mg/l 2,4-D showed the best callus formation with regeneration properties. Studies by Papafotiou and Martini^1^ and Vanzie-Canton and Leonhardt have confirmed the influential role of 2,4-D hormone in callus formation, which is in agreement with our results.

In this study, survival percentage and the regeneration rate of the *Zamiifolia* decreased with the increase in irradiation dose. The average PIC parameter for the primers of this study was calculated as 0.22 (0.06–0.47) depending on different germplasms. The number of alleles depends significantly on the sample size. Therefore, the sample size should be equal to obtain a significant comparison between samples due to many alleles at low frequencies in natural populations. In this study, the average number of alleles varied between 1.22 and 1.74. The lowest and highest number of effective alleles were observed in SWE and AK66 primers, respectively. Also, the highest genetic diversity here was observed in the AK66 gene locus, which showed the highest expected heterozygosity value in this study. The highest Shannon diversity index was also observed in the AK66 primer, which indicates that the AK66 primer shows the highest diversity performance in the genotypes. In this study, a high level of polymorphism was identified using ISSR markers and showed a high level of genetic diversity in the studied genotypes. Genetic relationships between studied genotypes were evaluated using ISSR marker and cluster analysis information. Calculating the Cophenetic correlation coefficient can demonstrate the performance of the used algorithm in converting similarity matrix to dendrogram. The Cophentic correlation coefficient(r = 0.7131 obtained from ISSR marker data) revealed that the Dice similarity coefficient and UPGMA algorithm were good methods for dendrogram drawing, meaning that the UPGMA algorithm has successfully converted the similarity matrix calculated by the Dice method into a dendrogram. According to the dendrogram, the genotypes can be classified into six groups. Therefore, the ISSR marker was able to classify the genotypes correctly.

## Conclusion

In the callus regeneration stage, the concentration of 0.5 mg/l BA and 0.5 mg/l NAA showed the highest number of regenerated seedlings and tubers. Due to the rooting of the tubers in the regeneration environment, rooting treatment was not necessary, and the seedlings easily adapted. The cocopeat and perlite substrate were suitable for the compatibility of *Zamiifolia* seedlings in the glass. The optimal dose of gamma irradiation, and LD_50_ was determined as 68 Gy for *Zamiifolia* irradiation. The diversity within the population of 15 parental, control, and irradiated genotypes was well shown by ISSR molecular marker and cluster analysis, which was generally grouped into three groups with low, medium, and high genetic diversity. Since *Zamiifolia* is one of the ornamental leaf plants without flowers and without the possibility to cross and follow the genetic diversity, the use of irradiation to create diversity in *Zamiifolia* is generally considered leading to successful results. Gamma rays caused variation in the shape of leaves and growth rate of plants, and the ISSR molecular marker test confirmed the variation in the population resulting from irradiation. Therefore, the following materials are suggested for *Zamiifolia* to continue the research:

1. Creating a large in vitro population of the *Zamiifolia* plant and inducing a dose higher than the optimal dose by gamma rays to increase the usefulness of irradiation and produce marketable cultivars.
2. Identifying specific markers in the population resulting from irradiation that are in linkage disequilibrium with commercial traits for quick screening of the population and follow-up of essential traits.
3. The use of living and non-living stresses such as the amount of light to identify and confirm promising *Zamiifolia* genotypes.
4. Simultaneous use of genetic engineering and genetic mutation techniques to produce new cultivars.

## Materials and methods

This study was conducted on parts of *Zamiifolia* leaves with the scientific name *Z. zamiifolia*. The *Zamiifolia* pots kept in the greenhouse of Ornamental Plants Research Center, Iran and Ornamental Plants Research center were used for tissue culture experiments, and all the experimental stages were carried out in the tissue culture laboratory of the commercial Royan Nahal Company, Iran.

### Tissue culture

The leaf parts were placed in 70% alcohol for 30 min after washing with distilled water and transferred to a container containing 1% hypochlorite with Tween 20 for 20 min. Then washing was performed in three steps (5, 10, and 15 min) under the laminar machine. The sterilized leaves parts were divided under the laminar into 1 × 1 cm pieces containing the main vein and cultivated in jars containing the culture medium^16^.

### Callus induction test

The basic culture mediums of this experiment to induce callus were MS, 2,4-D growth regulators at three levels (0, 1 and 2 mg/l) and BA at three levels (0, 1, and 2 mg/l)^16^.

### Regeneration test

After the emergence of callus and reaching the appropriate volume in terms of size and quality, all calluses were transferred to a new culture medium containing MS base culture medium for regeneration and plant production and then transferred together with NAA (0, 0.5 and 1 mg/l) and BA (0, 0.5 and 1 mg/l). The sucrose used was 30 g/l 1iter, and the pH of the medium was set to 5.7^16^. A factorial experiment based on a completely random design was used to determine the best treatments and their interaction effects. In this experiment, the plant growth regulators were investigated at nine levels in three replicates and 3–4 explants in each jar.

### Irradiation test

The calluses were sent to the Agricultural Atomic Energy Organization in Karaj, Iran for gamma-ray irradiation after optimizing callus induction, regeneration, and creating a certain number of *Zamiifolia* callus in terms of the highest rate of regeneration and determining the best treatment for regeneration. Irradiation under gamma rays was performed with ^60^Co sources with doses of 0, 10, 25, 50, 65, 75, 100, 125, 150, and 175 Gy in order to determine the optimal dose of gamma rays and then transferred to the callus induction environment after irradiation to prevent possible damage. In this regard, Sherpa et al.^17^ applied different gamma rays (10, 20, 40, 60 and 80 Gy) to induce mutagenesis and isolate mutants of Dendrobium (from Orchid flower family) for early flowering and phytomorphological traits. A total of 4 callus were cultured in each petri plate. Calluses were recorded in terms of survival rate after two weeks, and these data were used to determine LD_50_ dose. The calluses were irradiated with the optimal dose and transferred to the regeneration environment after the healing process. The explants were subculture every three weeks until regeneration and kept under controlled conditions. Finally, the irradiated calluses were transferred to the regeneration treatment culture medium.

### Storage conditions of cultivated explants

The storage conditions for callus culture and regeneration were 16 h of photoperiod (60 μmol/m^2^/s^2^) under the light of a white fluorescent lamp at 26 ± 2 °C.

### Compatibility of tissue culture plants

The in-glass plants obtained from the regeneration were transferred to the greenhouses of Royan Nahal Mahalat Company located in Mahalat village to check the possible diversity, and adaptation was made in the ratio of 1:1in the trays of seedlings in the cocopeat perlite substrate.

### Selection of promising genotypes

The morphological examination was carried out after the adaptation stages and seedling growth were passed. Each dose of leaf sample was transferred to the molecular markers laboratory of the Flower Ornamental Plants Research Institute for marker testing.

### Investigating diversity using molecular markers

DNAs were extracted using the Cetyl Trimethylammonium Bromide (CTAB) method, and 0.8% TBE was used to determine their quantity and quality from the nanodrop device and agarose gel. Optical absorption was read at 260, 280, and 230 nm wavelengths. Based on the melting temperatures of each pair of primers (supplementary Table 1), optimal PCR reaction conditions were selected and used for these primers. BioFACT™ 2X PCR Master Mix was used to perform the PCR reaction. A 0.2 mL reaction volume containing 10 µL of 2X PCR master mix, 1 µL of primer, 1 µL of diluted cDNA, and 8 µL double-distilled water to perform qPCR on an iCycler (BioRad 3600). The PCR conditions standardized in this study were an initial denaturation at 94 °C for five min; 35 cycles of denaturation (94 °C) for 30 s, annealing (55 °C) for 30 s, extension (72 °C) for one minute followed by final extension at 72 °C for 4 min. Agarose gel 1.5% was used to separate the amplified bands by PCR. For this purpose, 10 µl of PCR product along with two microliters of loading buffer loaded in gel wells and electrophoresis were performed. The integrity total RNA was assessed on the basis of visualization of 28S and 18S ribosomal RNA subunits under gel documentation system 2000 (Bio-Rad, Munchen, Germany).

### Data analysis

The results from the morphological traits were analyzed using SAS 9.1 statistical software, and the means were compared using Duncan's multi-range test at the 5% probability level. The figures were drawn using Excel software. Genetic analyzes were performed in the studied samples using GenALEx v6.5 and NTSYS v2.1 software. Bands were scored as presence (1) and absence (0), and then the presence of polymorphism in the mixture of different primers was investigated in all lines. On the base of the 0/1-matrix, a cluster analysis was conducted based on Dice similarity coefficients using unweighted pair group method with arithmetic average (UPGMA) and bootstrapping analysis was performed with 1000 re-sampling in order to test the reliability of the clusters. Moreover, the principal coordinated analysis (PCA) was done and the 2 principal coordinates were applied to visualize the differentiation of genotypes in a 2-dim array space. Dice similarity coefficient was used to prepare the similarity matrix, and clustering was conducted by UPGMA method. Genetic diversity indices, including polymorphism percentage, expected gene heterozygosity (He), effective allele numbers (Ne), and Shannon's information index (I), were evaluated using the GenAlEx software using the following formulas: $He = 1 - \sum\nolimits_{i = 1}^{n} {P_{i}^{2} }$, $Ne = 1/\sum\nolimits_{i = 1}^{n} {P_{i}^{2} }$, and $PIC = 1 - \sum\nolimits_{i = 1}^{n} {P_{i}^{2} } - \sum\nolimits_{i = 1}^{n - 1} {\sum\nolimits_{j = i + 1}^{n} {2P_{i}^{2} P_{j}^{2} } }$ , where *P*_*i*_ and *P*_*j*_ are the frequencies of *i*th and *j*th alleles and *n* is the number of alleles for the selected ISSR marker. The polymorphism information content (PIC), was calculated manually using Excel 2016.

## Supplementary Information

Supplementary Information 1.

Supplementary Table S1.

## Data Availability

The authors confirm that the data supporting the findings of this study are available within the article [and/or] its supplementary materials.
