# Supplementary material for: Evaluating the effect of gamma rays on Zamiifolia (Zamioculcas zamiifolia) plant in vitro and genetic diversity of the resulting genotypes using the ISSR marker
Source: Sci Rep. 2023 May 23;13:8308. doi: 10.1038/s41598-023-35618-2 (PMC10205732; doi:10.1038/s41598-023-35618-2)
Supplement: Supplementary file 2 — Supplementary Table S1. [file 41598_2023_35618_MOESM2_ESM.docx]

**Supplementary Table 1.** List of 22 ISSR primers

|  | Primer | Primer sequence (5’……3’) |
| --- | --- | --- |
| 1 | **OP1** | (TC)_8_C |
| 2 | **AK66** | (AC)_9_T |
| 3 | **SD7** | (TG)_7_GA |
| 4 | **LC26** | (ATG)_6_CT |
| 5 | **K1B** | (CTC)_7_C |
| 6 | **BCV3** | (GAA)_6_TT |
| 7 | **WB7** | (CCT)_8_A |
| 8 | **GN21** | (GA)_8_CT |
| 9 | **AVC1** | (CAG)_5_T |
| 10 | **AG5** | (CAA)_6_G |
| 11 | **SWE** | (AC)_8_TG |
| 12 | **PD12** | (CCG)_6_ |
| 13 | **SST** | (GACA)_4_ |
| 14 | **AHS** | (CA)_8_A |
| 15 | **CM2** | CTC(AG)_7_ |
| 16 | **F19** | (GAA)_6_C |
| 17 | **A86** | (CT)_8_A |
| 18 | **C07** | (AC)_8_G |
| 19 | **F20** | (GT)_8_CTC |
| 20 | **L19** | (TC)_8_AGA |
| 21 | **AN1** | (CCCT)_4_C |
| 22 | **C16** | (AC)_8_CTT |
